# OPA1, a molecular regulator of dilated cardiomyopathy

**DOI:** 10.1111/jcmm.17918

**Published:** 2023-08-21

**Authors:** Jiaqi Chen, Jianan Shao, Yaoyao Wang, Kangxiang Wu, Mingyuan Huang

**Affiliations:** ^1^ The Second Affiliated Hospital and Yuying Children's Hospital of Wenzhou Medical University Wenzhou China; ^2^ Fuwai Hospital Chinese Academy of Medical Sciences & Peking Union Medical College/National Center for Cardiovascular Diseases Beijing China

**Keywords:** apoptosis, cristae, dilated cardiomyopathy, fusion, OPA1, P53

## Abstract

Dilated cardiomyopathy (DCM) is a disease with no specific treatment, poor prognosis and high mortality. During DCM development, there is apoptosis, mitochondrial dynamics imbalance and changes in cristae structure. Optic atrophy 1 (OPA1) appears at high frequency in these three aspects. DCM LMNA (LaminA/C) gene mutation can activate TP53, and the study of P53 shows that P53 affects OPA1 through Bak/Bax and OMA1 (a metalloprotease). OPA1 can be considered the missing link between DCMp53 and DCM apoptosis, mitochondrial dynamics imbalance and changes in cristae structure. OPA1 regulates apoptosis by regulating the release of cytochrome c from the mitochondrial matrix through CJs (crisp linkages, located in the inner mitochondrial membrane) and unbalances mitochondrial fusion and fission by affecting mitochondrial inner membrane (IM) fusion. OPA1 is also associated with the formation and maintenance of mitochondrial cristae. OPA1 is not the root cause of DCM, but it is an essential mediator in P53 mediating the occurrence and development of DCM, so OPA1 also becomes a molecular regulator of DCM. This review discusses the implication of OPA1 for DCM from three aspects: apoptosis, mitochondrial dynamics and ridge structure.

## 1 INTRODUCTION

Dilated cardiomyopathy (DCM) is one of the causes of heart failure, arrhythmia and sudden death and is the most frequent indication for cardiac transplantation.^1^ Approximately 5–7 per 100,000 people occur DCM every year.^2^ DCM accounts for 20% of all heart failure diagnoses in the UK,^3^ and its treatment costs are estimated to be $4–10 billion per year in the United States.^4^ Data from 1982 showed that the 5‐year and 10‐year survival rates of patients with DCM were 54% and 36%, respectively.^5^ The mortality rate of DCM remains high despite advances in medical and interventional treatment‐survival rate at 10 years is approximately 60%.^6^ Because there is no specific treatment method and high mortality for DCM, it is urgent to find some active and effective treatment methods.

With the progress of research, it was found that DCM cardiomyocytes had apoptosis, imbalanced mitochondrial dynamics and changes in cristae structure. In the early 2000s, an increase in the number of apoptotic cells in cardiomyocytes was found in DCM, especially in advanced DCM.^7^ Biopsies and autopsy of patients with DCM revealed cardiomyocyte apoptosis in DCM. They were associated with worsening cardiac function, suggesting that apoptosis is essential for DCM development.^8^ Cardiac tissue from patients with DCM has a higher number of fragmented mitochondria.^9^ Studies in Mff‐deficient mice found that unbalanced mitochondrial fusion and fission mediate DCM.^10^ Eighty‐five cardiomyocyte biopsies had abnormal cristae and inclusion bodies in 601 patients with DCM.^11^ Autophagosomes containing abnormal mitochondria with swollen cristae were found in DCM cardiomyocytes in biopsies from 32 patients with DCM.^12^ Furthermore, OPA1 was involved in three aspects of apoptosis, mitochondrial dynamics imbalance and cristae structure.^13^, ^14^, ^15^

**FIGURE 1:**
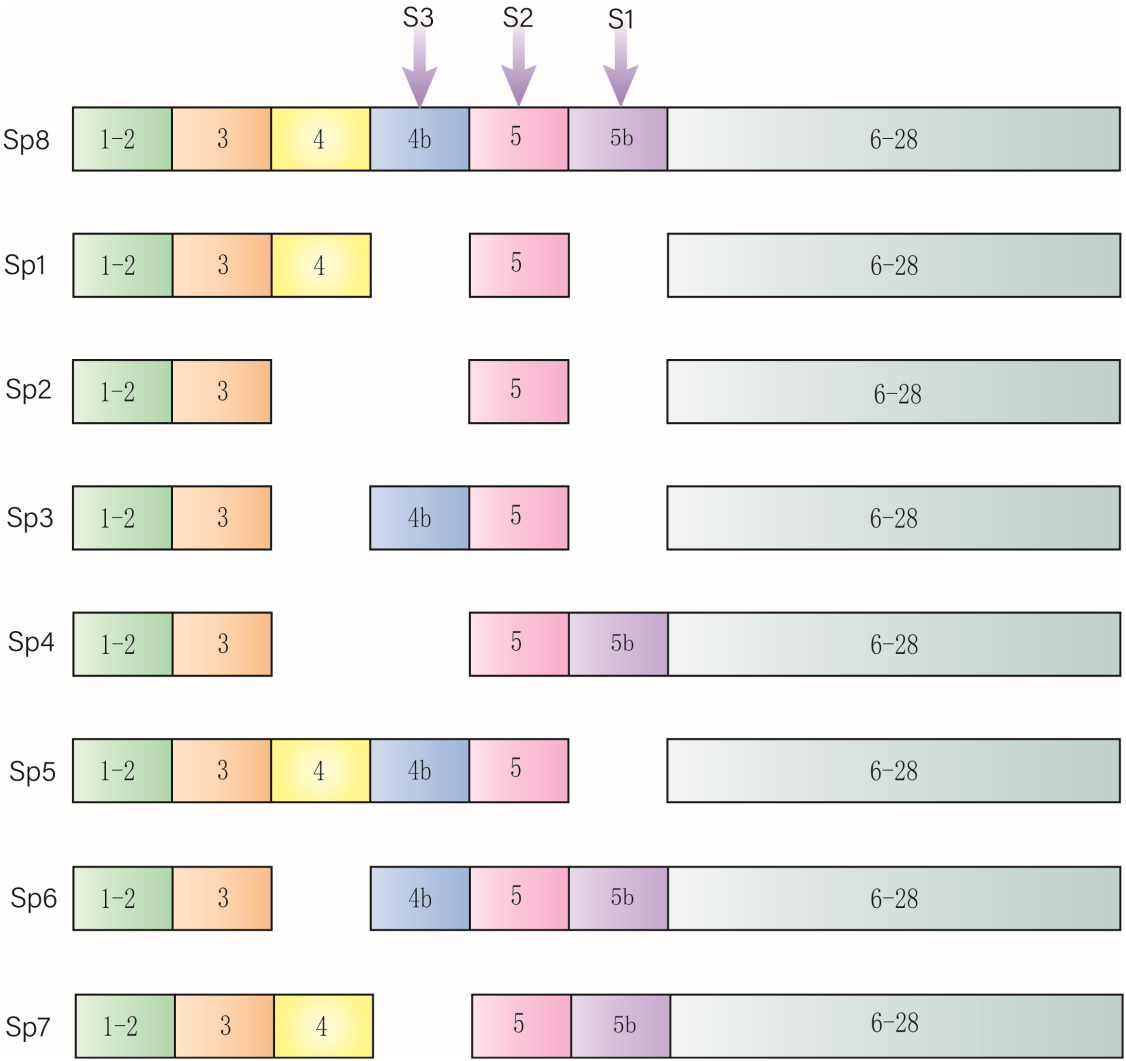
The OPA1 gene has 30 exons, numbering 1–28, 4b, 5b and splicing into eight kinds of mRNA. There are three hydrolytic sites S1, S2 and S3 in L‐OPA1, corresponding to 5, 5b and 4b, respectively.

Optic atrophy 1 (OPA1) belongs to the dynamics‐related proteins and has large GTP enzymes.^16^ It was initially found that OPA1 is associated with autosomal dominant optic atrophy, and the OPA1 gene is the most common mutation site of optic atrophy.^17^ The OPA1 gene is a single nuclear gene located at the end of chromosome 3q. The transcriptional product has 30 exons numbered 1–28, 4b, 5b and the latter two are vertebrate‐specific,^18^ transcribed into hnDNA and selectively spliced into eight kinds of mRNA^19^ (Figure 1). Human central expression subtypes 1 and 7 (Sp1 and Sp7)^20^, ^21^—the original translation products called OPA1‐DHFR. The activation of OPA1 is L‐OPA1 and S‐OPA1. It can be roughly considered that OPA1‐DHFR has a mitochondrial transit sequence (IMS) at the N‐terminal compared with L‐OPA1, which is degraded by MMP (mitochondrial processing peptidase) after transmembrane.^22^, ^23^ Compared with S‐OPA1, L‐OPA1 has a transmembrane structure (TM). OMA1(a metalloprotease) or Yme1L (a i‐AAA protease) acts on S1 and S2 sites between L‐OPA1 TM and G domain to generate 1–2 S‐OPA1, respectively (S2 is after S1, there is no sequential relationship between the two sites, and the two sites exist independently). They closely cooperate to maintain the balance of two OPA1 forms. Sp1 has only S1 site, while Sp7 has both, so human L‐OPA1 has three states: e (OMA1 hydrolysis), c (OMA1 hydrolysis) and d (Yme1L hydrolysis).^24^, ^25^, ^26^ The result is that L‐OPA1 is anchored in the inner membrane (IM), its N‐terminal location in the matrix, its C‐terminal location in IMS (intermember space). S‐OPA1, dissociated in IMS,^22^, ^26^ may be required for maintaining IMS homeostasis in mitochondria.^27^ Recently, it has also been found that the third hydrolysis of L‐OPA1 is site S3, mediated by Yme1L.^28^

## 2 OPA1 IS INVOLVED IN APOPTOSIS DURING DCM DISEASE PROGRESSION BY AFFECTING THE RELEASE OF CYTOCHROME C

The correlation between DCM and apoptosis has been confirmed with research progress. Mice with LMNA (LaminA/C) gene mutation (DCM model) has more than five times more apoptosis in cardiomyocytes than WT mice.^29^ TUNEL (DNA fragment marker) positive cardiomyocytes were found in 26 (86.7%) of 30 DCM patients with endomyocardial biopsy, and the apoptosis index (*p* < 0.001) in DCM was significantly higher than that in healthy controls.^30^

**FIGURE 2:**
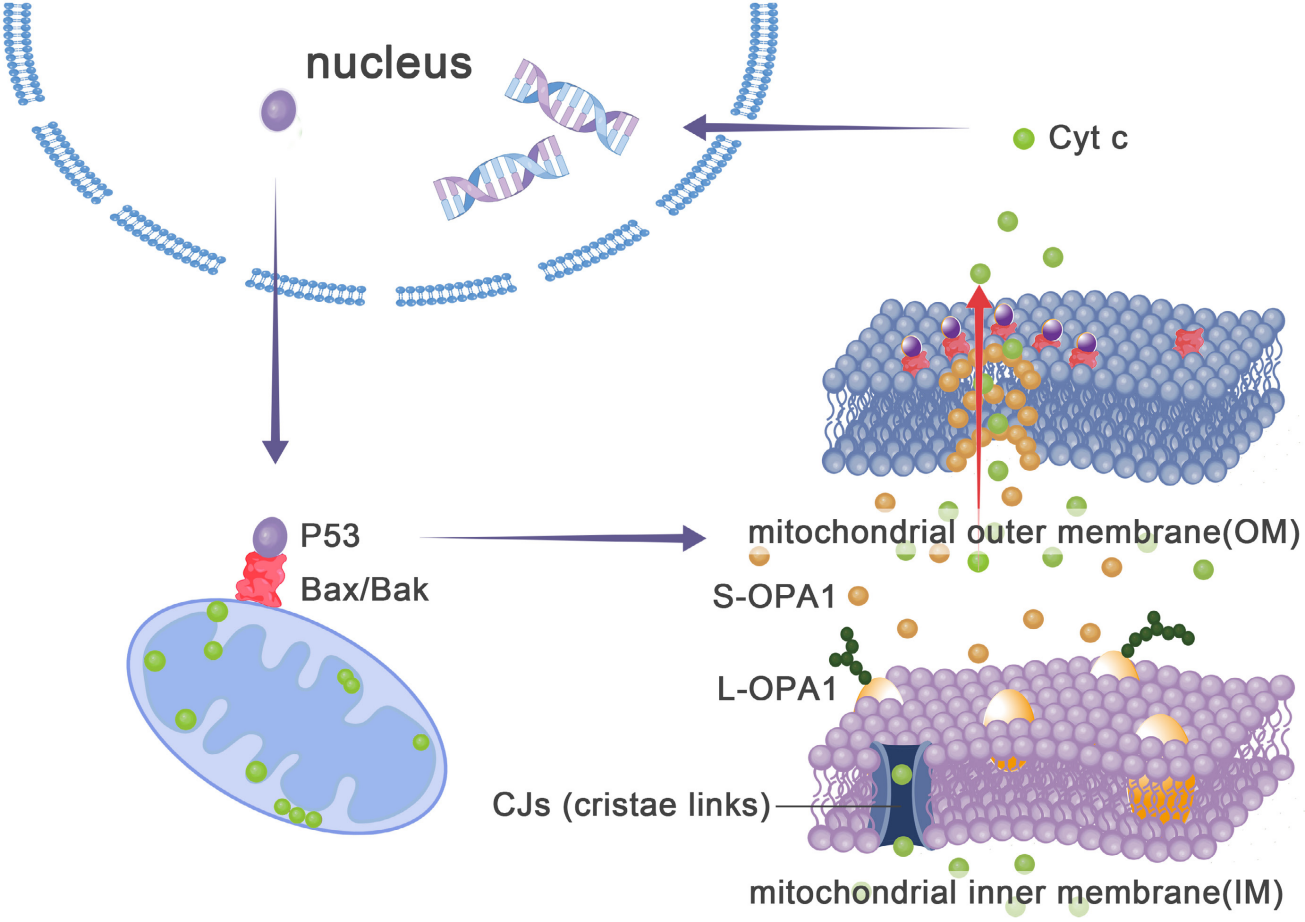
P53 leaves the nucleus and reaches the mitochondrial to activate Bak/Bax in OM, and Bak/Bax oligomerization activates OMA1. OMA1 hydrolysis of L‐OPA1 unbalances L‐ and S‐ OPA1, disintegrates OPA1 oligomers and opens CJs. At the same time, Bak/Bax oligomers on the OM form a ring structure, and pores are formed in the OM with the help of membrane‐shaking proteins such as S‐OPA1. Cytochrome c is released from the mitochondrial matrix into the cytoplasm through these two pores.

In the recent research on the pathogenesis of DCM caused by LMNA gene mutation, it was pointed out that the downstream mechanism of LMNA mutation in DCM is the E2F/DDR/TP53 pathway, and pointed out that the apoptosis of DCM is related to the activation of TP53.^29^, ^31^ The P53, TP53 gene product, was confirmed to be associated with apoptosis in cell experiments.^32^ It has been proved in tumour cell experiments that P53 can travel from the nucleus to the cytoplasm and then bind to Bcl‐2 (apoptosis inhibitor), inhibiting Bcl‐2 and activating Bax/Bak (members of the Bcl‐2 family and core regulators of the intrinsic pathway of apoptosis),^33^ and Bak activation is associated with p53 ser15 phosphorylation.^34^ Bax/Bak can act as the starting point of the caspase pathway apoptosis. Bax/Bak may affect the membrane potential (ΔΨ) by affecting the ion permeability of the inner mitochondrial membrane, and the change in ΔΨ can serve as the activation condition of OMA1. OMA1 activision mediates OPA1 processing, resulting in the unbalanced proportion of L‐opa1 and S‐OPA1.^24^ Changes in OPA1 have been demonstrated in p53‐bearing tumour cells.^33^, ^35^ However, this process is debatable. There is no detailed study that Bax/Bak must lead to the activation of OMA1 through ΔΨ and OMA1 must be activated under the change of ΔΨ. For example, antibiotic A changes Δψ, but does not affect OMA1 processing of OPA1, and oligomycin did not alter Δψ to activate OMA1.^24^ It has also been shown that OMA1 activation is associated with disrupting mitochondrial scaffolds by p53‐binding inhibin.^33^ OPA1 is involved in releasing cytochrome c (one of the markers of apoptosis) (Figure 2). In the experiment of downregulating OPA1 expression by specific siRNA, it was found that OPA1 is involved in the storage of cytochrome c, and it is speculated that OPA1 can be a target for controlling apoptosis.^36^ Cytochrome c release is increased in the absence or excess of OPA1.^37^ However, some studies have also found that a tiny quantity of OPA1 can reduce the release of cytochrome c.^38^ OPA1‐knockout HeLa cells undergo mitochondrial fragmentation followed by cytochrome c release and induction of apoptosis.^39^ Cytochrome c Centering the IMS from the matrix through OM by the small holes on the OM composed of Bax/Bak oligomerization. This process is regulated by the membrane‐shaping properties of excess S‐OPA1.^33^, ^40^, ^41^ Cytochrome c centering the cytoplasm affects DNA stability and mediates the process of apoptosis through pro‐caspase9 and pro‐caspase3.^42^

Cytochrome c is released from the IM in two ways: one is mitochondrial rupture and the other is the opening of CJs (cristae links, located at the junction of the mitochondrial cristae and boundary membrane), the latter being affected by OPA1.^40^, ^43^ The formation, structure and function of CJs are related to OPA1. Formation: Stephan and his colleagues found that the formation of CJs mainly depends on the Mic60 complex of MICOS (mitochondrial contact site and cristae organizing system). OPA1 and F1F0‐ATP synthase affect the localization of the MICOS complex and the stability of the CJs structure.^44^ Glytsou and his colleagues consider OPA1 to be the superior of Mic60.^45^ Structure: OPA1 deficiency did not affect the diameter of CJs under normal conditions. However, it was broadened under stress conditions, and the broadening process could be inhibited by overexpression of OPA1.^43^ The absence of OPA1 results in a decrease in the number of CJs and a widening of CJW (CJs width).^45^ Function: CJs function as channels, affecting the distribution of substances inside and outside the mitochondria.

Moreover, a certain proportion of L‐OPA1 and S‐OPA1 does not exclude other ingredients that formed OPA1 oligomers.^24^ The OPA1 oligomer is at least a trimer composed of two IM and one IMS OPA1.^43^ OPA1 oligomers act like a ‘gate’ to block CJs, preventing the release of small molecules such as cytochrome c (about 85% in the mitochondrial matrix) from the matrix to the IMS through CJs.^40^, ^43^, ^46^ By affecting the release of cytochrome C, OPA1 is a crucial substance in apoptosis during the progression of DCM and possible to regulate apoptosis during DCM progression.

## 3 OPA1 MEDIATES THE OCCURRENCE AND DEVELOPMENT OF DCM BY AFFECTING IM FUSION

There is an imbalance in mitochondrial dynamics during DCM development. The studies on the progression of autoimmune myocarditis to DCM induced by TLR4 (Toll‐like receptor 4) activation revealed an imbalance in mitochondrial dynamics involved in the pathophysiological process of DCM.^47^ Following an imbalance in mitochondrial dynamics in mouse cardiomyocytes, mouse cardiomyocytes become dysfunctional and develop dilated cardiomyopathy.^48^ End‐stage DCM is associated with abnormally enhanced fragmentation of the mitochondria.^49^ The activation of the TP53 pathway has been described in the LMNA gene mutation mimicking the DCM model. Still, there are few direct studies on the effects of TP53 activation on mitochondrial dynamics in DCM, but other studies cells have. Kong and his colleagues found that P53 regulates L‐OPA1 processing by affecting OMA1 in the study of tumour cell P53, affecting mitochondrial dynamics, and speculated that Bak/Bax acts as a ‘bridge’ between p53 and OMA1.^32^, ^35^ It is undeniable that there is an imbalance in mitochondrial dynamics in DCM development, which could be related to the downregulation of OPA1.^47^ However, a recent study has shown that the OMA1 gene is downregulated in studies of dilated cardiomyopathy, but there is no significant difference in OPA1 compared to the control group.^50^ Specific ablation of cardiac Yme1L in mice to activate OMA1 accelerates OPA1 proteolysis, triggering mitochondrial fragmentation and altering cardiometabolic, leading to dilated cardiomyopathy.^48^

**FIGURE 3:**
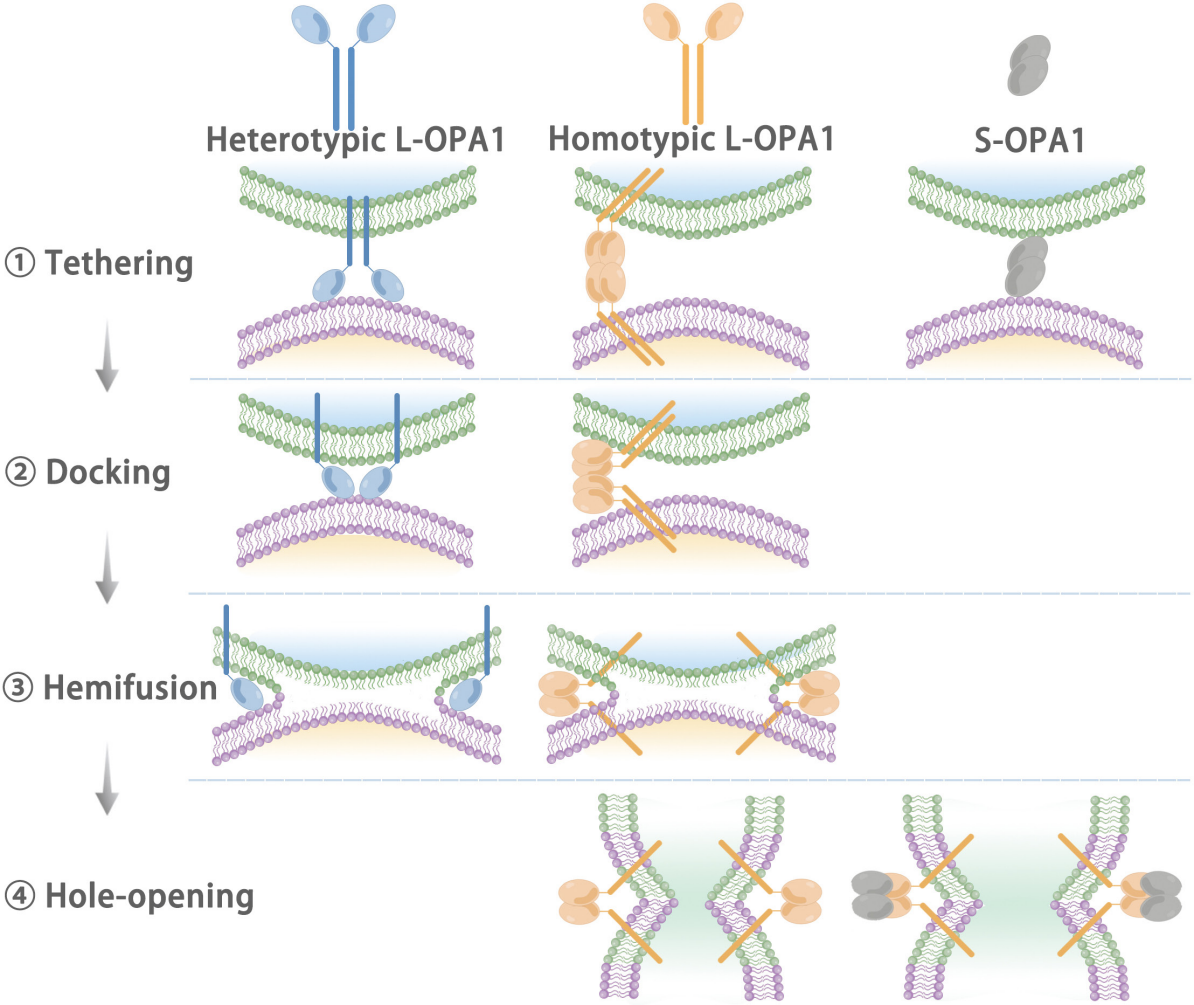
There are four steps in IM fusion: tethering, docking, hemifusion and hole opening. Among S‐opa1 lonely is capable of tethering but no docking and fusion; When heterotypic L‐OPA1 exists on one side, tethering, docking and hemifusion can be carried out alone, but hole opening cannot; Homotypic L‐OPA1 mediate complete four steps. Especially, S‐OPA1: L‐OPA1 is 1:1, and the efficiency of hole opening is the highest.

OPA1 is mainly involved in the IM fusion part of mitochondrial dynamics.^51^ A study on OPA1 disease‐causing mutants revealed that mutations inhibit mitochondrial fusion.^52^ The role of L‐OPA1 in IM fusion is rarely questioned. Studies with yeast Mgm1 (mitochondria genome maintenance 1), the mammalian OPA1 homologue, revealed that L‐Mgm1 promotes fusion and inhibits GTPase activity, while S‐Mgm1 hydrolyzes GTP to promote fusion.^53^, ^54^ The role of S‐OPA1 in the fusion process has been disputed.^55^, ^56^ Anand and his colleagues showed that L‐OPA1 is sufficient to mediate IM fusion in Yme1L‐ and OMA1‐deficient cells, while S‐OPA1 is involved in mitochondrial fission.^55^ Some studies have also suggested that both L‐ and S‐ OPA1 are required in mitochondrial fusion, but S‐OPA1 cannot play a role in the fusion process alone.^57^, ^58^, ^59^ S‐OPA1 is necessary for fusion in studies that inhibited conversion to S‐OPA1 by mutations in L‐OPA1 while also acknowledging that it also plays a role in the division.^24^, ^56^ Recent research has also shown that mitochondrial morphology is highly sensitive to the ratio of L‐OPA1 to S‐OPA1, indicating that mitochondrial fusion is regulated by S‐OPA1.^28^ As research progresses, most studies agree that L‐ and S‐ OPA1 play respective roles in the four steps of IM fusion (tethering, docking, hemifusion and hole opening)^16^ (Figure 3). Tethering: Ban and his colleagues proposed that L‐OPA to cardiolipin (CL) does not depend on GTP to mediate tethering, and L‐OPA1 on one side can mediate fusion (under normal circumstances, both sides have L‐OPA1 in the fusion process). This process is called ‘Fusion Mode.’ S‐OPA1 can act as a bridge to improve fusion efficiency.^19^, ^57^, ^60^ However, Ge and his colleagues, combined with the research results of Faelber'team, speculated that L‐OPA1 to CL may be mediated by Paddle (one of the four significant OPA1 domains), and CL acts as a dependent factor in the tethering process.^61^ Their results suggest that tethering can be mediated by homotypic L‐OPA1 to L‐OPA1, and this process does not require GTP, and CL is its dependent factor. S‐OPA1 alone can also play the role of tethering and can be enhanced by GTP. Docking: Heterotypic L‐OPA1 on one side can mediate effective docking and not affect GTP. Homotypic L‐OPA1 needs GTP for efficient docking and requires both sides. S‐OPA1 cannot mediate docking, but it may play other roles, such as regulating docking. Hemifusion: homotypic L‐OPA1 mediates hemifusion better than heterologous L‐OPA1. The vital step in the IM fusion process is hole opening. Homotypic L‐OPA1 is sufficient to mediate hole opening, while heterotypic L‐OPA1 and S‐OPA1 do not directly. Further studies found that S‐OPA1 regulates hole‐opening efficiency, especially at the equal molar time of L‐OPA1 and S‐OPA1. If homotypic L‐OPA1 tethering is used as the starting point, about 8% will enter full integration.^16^ The latest research finding is that GTPase activity is necessary in mitochondrial fusion, but GED (GTPase effector domains) is dispensable.^52^ Due to GTP‐anchored L‐OPA1 impairs GTPase activity, the energy during fusion comes from the hydrolysis of S‐OPA1.^57^ OPA1 participates in mitochondrial dynamics by influencing IM fusion, which plays a role in DCM's occurrence and development and provides a new direction for DCM treatment.

## 4 THE ABNORMALITY OF MITOCHONDRIAL CRISTAE IN DCM MAY BE RELATED TO OPA1

With the advent of electron microscopy (EM), the ultrastructure of DCM has gradually become apparent. Ten biopsies of cardiomyocytes from patients with DCM were randomly selected for ultrastructural assessment. It was found that the size, shape and distribution of mitochondria were different from normal cardiomyocytes. The mitochondrial structure was blurred and the mitochondrial cristae were partially lost.^62^The mouse control experiment found that the number of mitochondria in the cardiomyocytes of mice in the DCM group increased abnormally, aggregated into clusters, and appeared highly swollen, cristae fractured or disappeared, and vacuolized.^63^ The cristae were once thought to be static, but with the development of science and technology, it is gradually recognized that they are highly dynamic.^64^, ^65^ However, this discovery makes studying the maintenance and formation of related substances and their functions in the original exploration is more complicated. Many studies regulate mitochondrial cristae formation and maintenance, but there is no clear answer. It is generally accepted that mitochondrial cristae are related to OPA1, ATP synthase and the MICOS complex.^66^, ^67^, ^68^, ^69^ The conclusion that cristae formation and maintenance are associated with OPA1/Mgm1 initially arose from the discovery that OPA1/Mgm1 loss was related to impaired respiration and growth.^70^, ^71^ After the loss of OPA1/Mgm1 under EM, the cristae were almost absent, and the whole mitochondria were almost empty or only some septum.^72^, ^73^

Harner and his colleagues found that yeast Mgm1 is directly involved in cristae formation but is limited to lamellar cristae (the heart has two main mitochondrial cristae morphologies, a lamelliform located in the subsarcolemmal and a tubular located in the interfibrillar^74^). There are two forms, one is that two IMs fuse into lamellar cristae during the fusion process, which requires Mgm1, F1F0‐ATP, MICOS comlex and forms CJs to limit fusion, and the other is that the formation of tubular crest does not require Mgm1, only requires F1F0‐ATP and MICOS comlex.^72^ The former is essentially the fusion of two membranes, while the latter is more similar to folding too‐long membranes. Mgm1 is a homologue of OPA1. Although the structure is conserved, whether mammalian OPA1 plays the same role in cristae formation remains further investigated. Hu et al.' study pointed out the diversity of cristae and the formation pathway of multiple cristae. The abnormal cristae fusion study pointed out the form of cut through cristae and spherical cristae, and cut through cristae can eventually be transformed into lamellar crista.^67^ Ban and his colleagues proposed that membrane fusion‐related ‘cristae pattern’ mediates cristae formation, which is tethering and cristae formation mediated by the homotypic L‐OPA1.^57^ Stephan and his colleagues found that the lamellar cristae formed a group and speculated that the cristae of tissue originated from a membrane precursor. It is worth mentioning that the cristae formation had nothing to do with the fusion or division of OM (mitochondrial inner membrane).^44^, ^75^

Gene ablation or knockout or otherwise affecting OPA1/Mgm1 in earlier studies also resulted in abnormal cristae structure.^36^, ^43^, ^76^, ^77^ In recent research on neurons, it has also been found that inactivation of OPA1 causes dramatic alterations in cristae topology.^78^ Downregulation of OPA1 by siRNAs leads to cristae disintegration.^36^ Overexpression of OPA1 can increase the number of cristae and reduce the cristae width (CLW), suggesting that OPA1 is required to maintain cristae integrity.^38^, ^76^ Glytsou and his colleagues state that CLW is mainly regulated by OPA1.^45^ Further research has found that elongated mitochondria with a notable stacking phenotype and an absence of tubular cristae are observed when mitochondria contain mainly L‐OPA1. However, irregular cristae packing and an increase in globular cristae are found when S‐OPA1 is mainly present.^79^ It was found that OPA1/Mgm1 primarily exists in the cristae and is not much distributed near CJs,^77^ but there is no thorough study on how OPA1 plays its role at the molecular level. When summarizing the relationship between OPA1\Mgm1 and cristae structure, LenaPernas et al. came up with such a model: L‐OPA1 oligomers will be distributed along the cristae, maintaining the tethering between the cristae gaps, thus affecting the cristae structure.^80^ However, whether S‐OPA1 should be involved in this oligomer like the OPA1 oligomer in CJs, and whether this model is valid are still unsolved topics. It is worth mentioning that the formation of cristae curvature is less related to Mgm1/OPA1 and more pertaining to MICOS comlex.^81^ Overall, OPA1/Mgm1 in cristae formation and maintenance of cristae structure is beyond doubt. LMNA mutation to TP53 activation certainly exists in DCM. The effect of p53 on L‐OPA1 was also confirmed in tumour cells. However, there are few direct studies on the cleavage of DCM from P53 to L‐OPA1, and there is still room for further research.

**FIGURE 4:**
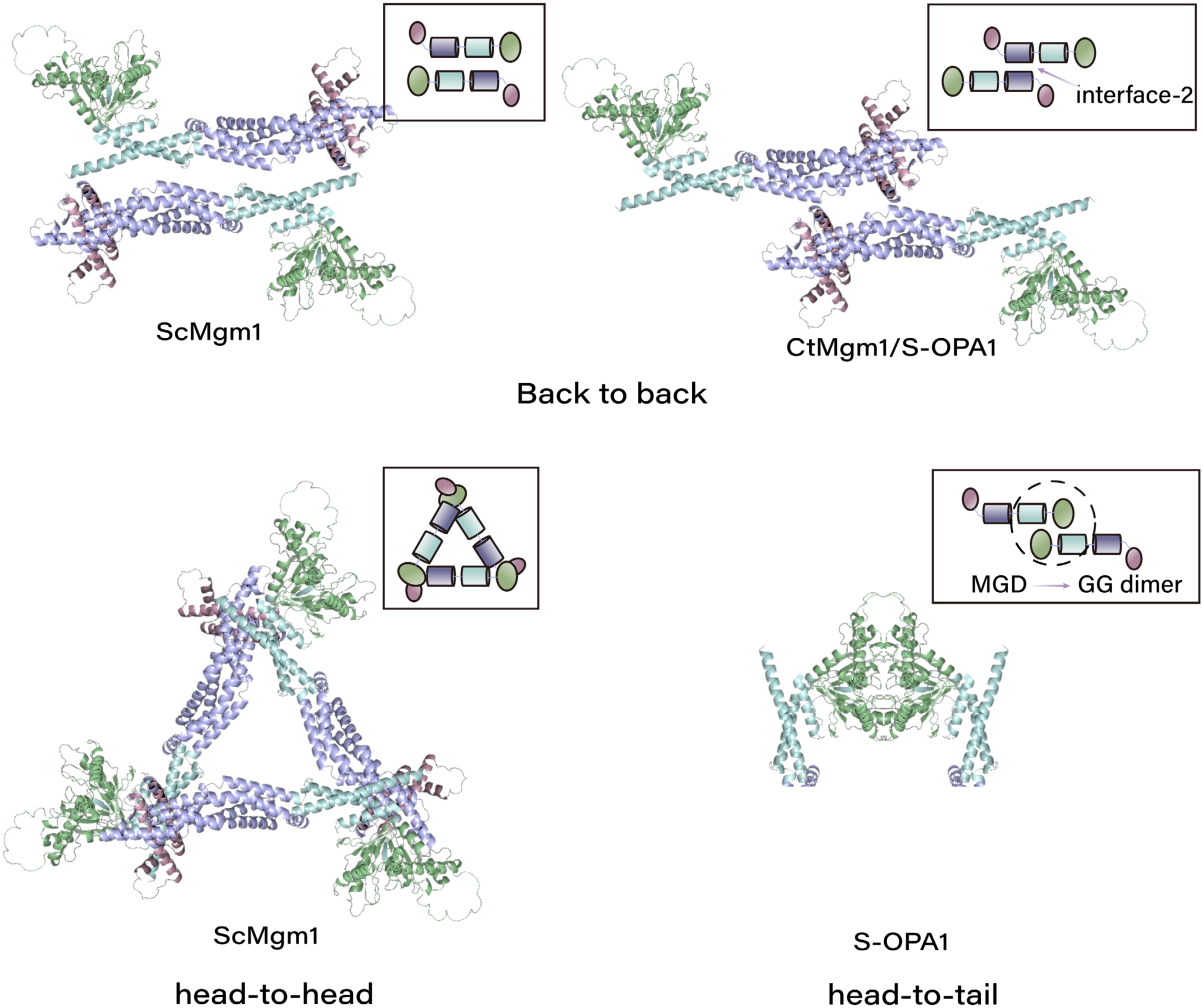
S‐OPA1/S‐Mgm1 consist of a G domain (green), a bundle signalling element (BSE) /BH1 domain (blue), a stalk/BH2 (purple) and a paddle/LIS domain (pink). The Schematic also shows the structural assembly diagram of Saccharomyces cerevisiae (Sc) Mgm1, Chaetomium thermophilum (Ct) Mgm1, and OPA1 MGD. The dimer is formed through the interface‐2 between the opposing stalks. Furthermore, the interaction between the G domain and stalk‐paddle forms the trimer. MGD, composed of the G domain and BSE, two asymmetric OPA1‐MGD form GG dimer.

The role of L‐OPA1 in cristae is generally affirmed, while the part of S‐OPA1 is often denied.^55^, ^56^ However, a recent study found that S‐OPA1 is sufficient to form and maintain cristae structure in OPA1‐deficient cells. Lee and his colleagues have found that S‐OPA1 can maintain the cristae structure and function in the absence of L‐OPA1 and consider that mitochondria may be affected by the additional effect of OMA1/Yme1L removal.^56^ S‐Mgm1 and S‐OPA1 induced liposome membrane tube formation and modification, which had nothing to do with GTP. Membrane tube formation increased GTP hydrolytic activity of S‐OPA1.^61^, ^82^ Mgm1/OPA1 oligomers mediate liposomal membrane tube formation similar to that of split DLPs,^83^, ^84^ but the structure of membrane tubes resembles cristae.^61^ Gao and his colleagues considered that S‐Mgm1/S‐OPA1 might wrap the protruding IM, induce cristae formation and affirm that the back‐to‐back structure of S‐Mgm1/S‐OPA1 is necessary for cristae formation.^85^ Stalk (one of the OPA1 domains) mediates the ‘back‐to‐back’ structure of S‐Mgm1/S‐OPA1 (Figure 4). Chaetomium thermophilum (Ct) Mgm1 forms a dimer, tetramer and alternately filament through stalk interface‐2 and stalk interface‐1. Four parallel helical filaments further form a left‐handed four‐start helix that modified the membrane tube's outer surface and inner surface by Paddle. When CtMgm1 is combined with GTP, the membrane tube on the outer surface modified by the left‐handed spiral expands and shrinks on the inner surface.^61^ Compared with CtMgm1, S‐OPA1 forms a dimer through interface‐2, interacts with EMB domain interface P1 (located in Paddle) through stalk interface‐3 in dimer units, and is further stabilized by interface‐1 between the same ladder, forming a helix assembly with six‐helix starting points. Compared with CtMgm1, there are more interface‐3 and P1. The structure is spirally assembled to the membrane through P1, which is considered a ‘closed conformation.’ When it combined with GTP, interface‐3 and P1 are broken. Different steps (adjacent to fragment) through the EMB P2 interaction form a new ladder, thought to be an ‘open conformation,’ where the spiral tube expands, and the membrane tube expands, but GTP is not hydrolyzed.^82^ In addition to the ‘back‐to‐back’ structure, the ‘head‐to‐tail’ trimer structure of S‐Mgm1 in Saccharomyces cerevisiae (ScMgm1) is mediated by the interaction between G domain and stalk‐paddle (Figure 4). It binds to the membrane through the G domain and Paddle, which induces the bending of the membrane to form an unstable membrane tip presumed to be related to the fusion of the membrane.^86^ This structure may affect the action of GTPase, whether it can be widely used in fusion remains to be discussed, and it has little effect on cristae. It has only been found in Saccharomyces cerevisiae so far.^85^ The widely existing structure in DLP is G‐G‐mediated ‘head‐to‐head’ structure^87^ (Figure 4). MGD, Minimal G domain, consists of G domain and BSE. Two mismatched OPA1‐MGD form GG dimer and each G domain has a GTP enzyme. Nucleotides are required in this process from structural analysis, and the presence or absence of nucleotides from the biochemical analysis can be. After the dimer formation, the local lipid concentration near OPA1 was increased, and then S‐OPA1, like S‐Mgm1, combined with IM to stimulate the hydrolysis of GTP. Whether this structure is related to the membrane tube has not been definitively concluded. Gao and his colleagues believe that it may play an essential role in cristae,^85^ but there is room for further investigation.

## 5 CONCLUSION AND PROSPECT

In summary, it is speculated that there is a P53‐Bak/Bax‐OMA1‐OPA1‐like relationship axis in DCM. After the LMNA gene mutation, the TP53 pathway is activated, and P53 goes from the nucleus to the cytoplasm to activate Bak/Bax, which may trigger much OMA1 self‐cleavage maturation through membrane potential changes. Or p53 binds inhibin activating OMA1 activation by disrupting mitochondrial scaffolds. M‐OMA1 hydrolyzes L‐OPA1 to unbalance L‐ OPA1 and S‐OPA1, affecting apoptosis, mitochondrial dynamics and cristae structure, thereby mediating the occurrence and development of DCM. These processes have been widely studied on various cells. Curcumin (an anticancer agent) was found to promote tumour cell apoptosis by activating p53.^88^ Protein tyrosine phosphatase 1B inhibition improves mitochondrial dynamics by regulating OPA1 homeostasis to alleviate calcified Aortic valve disease.^89^ Epigallocatechin gallate can inhibit the self‐cleavage of OMA1 and attenuate the cleavage of OPA1, thereby protecting cardiomyocytes from hypoxia‐reperfusion injury.^90^ Using NS1619 and dehydroepiandrosterone (DHEA) to increase mitochondrial reactive oxygen species (ROS) can activate OMA1 and affect OPA1 to kill T‐cell acute lymphoblastic leukaemia cells selectively.^91^ However, there are very few studies on the relationship between P53‐Bak/Bax‐OMA1‐OPA1 directly on DCM and how to influence DCM by affecting these relationships, which also provides direction for DCM research.

## AUTHOR CONTRIBUTIONS

**Jiaqi Chen:** Conceptualization (equal); methodology (equal); writing – original draft (equal). **Kangxiang Wu:** Methodology (equal). **Yaoyao Wang:** Methodology (equal); visualization (equal). **Jianan Shao:** Writing – review and editing (equal). **Mingyuan Huang:** Conceptualization (equal); writing – review and editing (equal).

## CONFLICT OF INTEREST STATEMENT

The authors state that there is no conflict of interest or competing interests.

## CONSENT FOR PUBLICATION

All the authors give consent.

## Data Availability

Data sharing is not applicable to this article as no new data were created or analyzed in this study.
